# Pathophysiology of Coagulation and Emerging Roles for Extracellular Vesicles in Coagulation Cascades and Disorders

**DOI:** 10.3390/jcm11164932

**Published:** 2022-08-22

**Authors:** Houssam Al-Koussa, Ibrahim AlZaim, Marwan E. El-Sabban

**Affiliations:** 1 Department of Pharmacology and Toxicology, Faculty of Medicine, The American University of Beirut, Beirut P.O. Box 11-0236, Lebanon; 2 Department of Biochemistry and Molecular Genetics, Faculty of Medicine, The American University of Beirut, Beirut P.O. Box 11-0236, Lebanon; 3 Department of Anatomy, Cell Biology and Physiological Sciences, Faculty of Medicine, The American University of Beirut, Beirut P.O. Box 11-0236, Lebanon

**Keywords:** coagulation, history of coagulation, extracellular vesicles, exosomes

## Abstract

The notion of blood coagulation dates back to the ancient Greek civilization. However, the emergence of innovative scientific discoveries that started in the seventeenth century formulated the fundamentals of blood coagulation. Our understanding of key coagulation processes continues to evolve, as novel homeostatic and pathophysiological aspects of hemostasis are revealed. Hemostasis is a dynamic physiological process, which stops bleeding at the site of injury while maintaining normal blood flow within the body. Intrinsic and extrinsic coagulation pathways culminate in the homeostatic cessation of blood loss, through the sequential activation of the coagulation factors. Recently, the cell-based theory, which combines these two pathways, along with newly discovered mechanisms, emerged to holistically describe intricate in vivo coagulation mechanisms. The complexity of these mechanisms becomes evident in coagulation diseases such as hemophilia, Von Willebrand disease, thrombophilia, and vitamin K deficiency, in which excessive bleeding, thrombosis, or unnecessary clotting, drive the development and progression of diseases. Accumulating evidence implicates cell-derived and platelet-derived extracellular vesicles (EVs), which comprise microvesicles (MVs), exosomes, and apoptotic bodies, in the modulation of the coagulation cascade in hemostasis and thrombosis. As these EVs are associated with intercellular communication, molecular recycling, and metastatic niche creation, emerging evidence explores EVs as valuable diagnostic and therapeutic approaches in thrombotic and prothrombotic diseases.

## 1. Introduction

Hemostasis is a dynamic physiological process, which controls bleeding at the site of injury while maintaining normal blood flow within the body. This occurs through the induction of blood coagulation, a process by which the activation of clotting factors forms a blood clot that halts bleeding [1]. The concept of blood coagulation was recognized as early as ancient Greece. However, Marcello Malpighi, an Italian scientist, observed and documented the process of blood coagulation in the 1600s. The surge in interest in this field resulted in the discovery of several coagulation factors, and accordingly, numerous theories were presented. As such, the theory of hemostasis became well refined and understood during the twenty-first century. The complex regulation of coagulation factors in hemostasis relies on the interaction among a multitude of effectors such as platelets, blood vessels, and other coagulation factors. Coagulation is coordinated by pro-coagulation factors that are countered by the process of fibrinolysis, which comprises a set of anti-coagulation factors and inhibitors regulating the coagulation cascade and maintaining hemostasis while preventing thrombosis [2,3]. However, states of hypo-coagulation and hyper-coagulation emerge secondary to defects in the coagulation cascade leading to either abnormal bleeding or abnormal clotting [4].

An exciting novel opportunity in studying coagulopathies is the implication of extracellular vesicles (EVs), in the modulation of coagulation processes [5,6,7,8]. EVs comprise micro-vesicles (MVs), exosomes, and apoptotic bodies. Studies on the mechanisms of EV formation, cargo sorting, and release have witnessed a surge in the literature. Importantly, platelet-derived EVs are highly implicated in the regulation of coagulation physiology and the pathophysiology of hypercoagulable states. Here, we present a historical background of coagulation up to the discovery of the cell-based model. We also describe the process of hemostasis from injury occurrence to blood clot dispersion. Importantly, we focus on the emerging evidence implicating cell-derived and platelet-derived EVs in the modulation of hemostasis and coagulopathies.

## 2. Coagulation: A Historical Overview

Theories of blood clotting date back to ancient Greece when the words hema (blood) and thrombus (clump) were introduced [9,10]. In the seventeenth century, novel concepts regarding coagulation deviating from the “traditional Galen medicine” were tabooed, contested, and disputed. However, Dr. Marcello Malpighi championed these revolutionary theories and identified, using a single lense microscope, that blood consists of a combination of red blood cells and a meshwork of fibrous material, now referred to as fibers [3,11]. In the early 1720s, Jean-Louis Petit, a French surgeon, was the first to link coagulation with hemostasis after observing patients healing from limb amputation, after whom Dr. William Hewson, a young British surgeon, observed that blood coagulum arose from the liquid part of the blood, which is now known as plasma. Moreover, he discovered that the plasma contains different substances like albumin, globulin, and the precursor of fibrin [12,13]. In 1801, Fourcroy introduced the term “fibrin” and demonstrated that its precursor exists in the plasma. Four decades later in 1847, Virchow identified the precursor of fibrin and named it “fibrinogen” [14]. In 1872, Alexander Schmidt’s research showed that an enzymatic process leads to the conversion of fibrinogen into fibrin, and he called the enzyme “thrombin”. Schmidt realized that thrombin cannot exist in its active state in the blood, thus he postulated the presence of a precursor, prothrombin [15]. These discoveries laid the foundation of Paul Morawitz’s classic theory of coagulation in 1905, whereby the schema of coagulation consisted of four factors “prothrombin, thrombin, fibrinogen, and fibrin” in the presence of calcium [2,16].

This classic theory of coagulation persisted until Paul Owren introduced modern insights on the biochemical processes of coagulation in 1947 [13], when he identified new coagulation factors including von Willebrand factor (vWF) [17], factor five (FV) [18], FVII [19], FVIII [20], FIX [21], FXI [22], and FX [23]. In 1964, Macfarlane proposed the cascade model, which introduces the order in which the clotting factors are activated. This model was followed by the waterfall model by David and Ratnoff in which each clotting factor is a proenzyme (zymogen) awaiting predecessor-mediated proteolytic cleavage to initiate its activity thus preventing indiscriminate activation or degradation [24,25,26]. Coagulation can be initiated by two different pathways; the intrinsic pathway secondary to vascular endothelium damage and sub-endothelial collagen exposure and the TF-dependent extrinsic pathway. However, the initiation of either pathway results in the activation of FX of the cascade, leading to the formation of a fibrin clot [27].

In 1935, Henrik Dam discovered that vitamin K was an important player in coagulation [28]. Indeed, vitamin K plays a role in the activation of prothrombin, factors VII, IX, and X, and the production of proteins C, S, and Z [29]. Another key player in coagulation is calcium, which is required for the conversion of prothrombin into thrombin [13]. Recent work on coagulation led to the development of a new model “the cell-based model” in which the exposure of cells expressing TF on their surface initiates blood coagulation in vivo. This model pinpoints that the intrinsic pathway does not have a proper physiological role in hemostasis, adjusts the role of FXII, and highlights its contribution to pathological thrombosis [30,31].

## 3. Hemostasis

**Figure 1:**
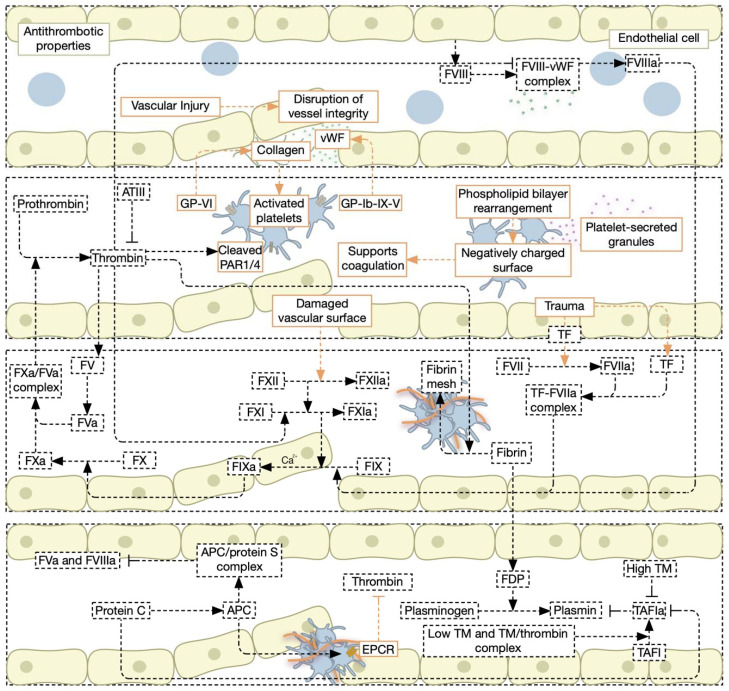
The hemostatic pathways include the intrinsic and the extrinsic coagulation pathways. In the healthy state, endothelial cells maintain an antithrombotic property that ensures normal blood flow. Following vascular injury and the disruption of vascular endothelial integrity, FXII is activated by proteolytic cleavage resulting in the formation of FXIIa. FXIIa activates FXI into FXIa, which then activates FIX into FIXa in the presence of calcium. FIXa activates FX into FXa which binds thrombin-activated FVa forming the FXa/FVa complex that activates prothrombin into thrombin. Then, thrombin cleaves fibrinogen into fibrin forming the fibrin mesh and causing the cessation of blood loss. Thrombin also cleaves PAR1/4 and activates FV into FVa and FXI into FXIa, thus reinforcing the coagulation cascade. Thrombin also inhibits the binding of the endothelial-derived FVIII to exposed vWF and thus, accelerates the formation of the FVIII/vWF complex and the subsequent activation of FVIII into FVIIIa, which activates FIX into FIXa. Additionally, thrombin activity can be inhibited by ATIII. Parallel to the intrinsic pathway, the extrinsic pathway is activated in response to vascular trauma, which results in the secretion of TF. TF activates FVII into FVIIa and subsequently forms the TF-FVIIa complex, which activates FIX into FIXa. Activated platelets play a major role in the potentiation of both pathways by providing negatively charged surfaces following phospholipid bilayer rearrangement, which supports coagulation. Following primary and secondary hemostasis, fibrinolysis takes place. Fibrin degradation leads to the formation of FDP, which activates plasminogen into plasmin. TAFI, which is activated by low TM and the TM/thrombin complex into TAFIa, inhibits plasmin activity. TAFIa is inhibited by both high TM and protein C. Finally, protein C activates APC, which binds to protein S forming the APC/protein S complex, which inhibits the activity of FVa and FVIIIa. Abbreviations: APC, active protein C; ATIII, anti-thrombin III; EPCR, endothelial cell protein C receptor; F, factor; FDP, fibrin degradation products; GP, glycoprotein; PAR, protease-activated receptor; TAFI, thrombin-activatable fibrinolysis inhibitor; TF, tissue factor; TM, thrombomodulin; vWF, von Willebrand factor.

Hemostasis is achieved through an intricate balance between the levels of procoagulant and anti-coagulant factors [32,33]. Blood vessels are lined by endothelial cells that exhibit intrinsic antithrombotic properties. However, secondary to vascular injury, the sub-endothelial matrix comprising collagens, VWF, and adhesion proteins, makes the environment highly thrombogenic to blood [34]. Hemostasis comprises primary and secondary phases that culminate in the formation and stabilization of fibrin strands forming the platelet plug [35]. Key hemostatic mechanisms are described in Figure 1.

### 3.1. Primary Hemostasis, Secondary Hemostasis, and the Cell-Based Model of Coagulation

During primary hemostasis, platelets and vWF accumulate rapidly at the sub-endothelial matrix following endothelial damage. Platelet adhesion to collagen is facilitated by injured tissue-released vWF. This occurs following platelet activation and the interaction of platelet surface glycoproteins receptors GPIb-IX-V and GPVI with immobilized vWF, and sub-endothelial collagen, respectively [35,36]. Both receptors are critical for platelet adhesion to the sub-endothelial matrix at the site of injury [37,38,39,40,41]. Activated platelets exhibit increased activity of surface integrins, αIIbβ3, α2β1, and αvβ3, that ensure platelet-platelet interactions, as well as their binding to collagen, VWF, fibrinogen, and fibronectin [42,43]. Moreover, the platelet phospholipid bilayer membrane is rearranged following platelet activation and becomes negatively charged to support the coagulation cascade later during secondary hemostasis [44]. The platelet membrane acts as a surface for the assembly of activated coagulation cascade during secondary hemostasis.

Concurrently, and during secondary hemostasis, extravascular cells including smooth muscle, fibroblastic, and endothelial cells, release TF (also known as FIIIa). Platelet-released polyphosphates activate FVII into FVIIa, which then binds TF forming the FVIIa/TF complex. In the presence of calcium, the FVIIa/TF complex activates FX into FXa, which consequently activates prothrombin into thrombin in the presence of its cofactor FVa [45,46]. In the extrinsic pathway, FXII is activated into FXIIa by the negatively charged membrane phospholipid surface of the platelets. FXIIa then cleaves FXI producing FXIa, which activates, in the presence of calcium, FIX to FIXa. FIX can also be activated by FVIIa through the intrinsic pathway. FVIII is a cofactor produced by endothelial cells and hepatocytes and is found as a complex with circulating vWF. Following vascular injury, FVIIIa dissociates from the complex with vWF and interacts with FIX, forming a new complex in the presence of calcium ions and negatively charged membrane phospholipids that activate FX into FXa [47,48].

The extrinsic and intrinsic pathways culminate at FXa which activates the serine protease thrombin, which cleaves fibrinogen into fibrin [49]. Thrombin further activates platelets through the proteolytic processing of the G-coupled membrane receptors, protease-activated receptor 1 (PAR1) and PAR4 [50]. Thrombin also activates FXI, which later activates FIX through the intrinsic pathway; but also FVIII and FV, creating a positive feedback activation loop for coagulation [49].

More recently, a cell-based model of coagulation was proposed implicating elaborate interactions between platelets, vessel walls, and the coagulation system, and better describes the in vivo coagulation processes [34]. This model comprises three distinct phases; initiation, amplification, and propagation rather than independent pathways [31]. An example of such intricate processes includes FXa interaction with FVa, which produces limited amounts of thrombin that plays a key role in the activation of FXI, FVIII, FV, and platelet PARs during amplification [30,51,52,53]. Following its activation by thrombin, FVa is released from the fully-activated platelets and interacts with FXa (activated by the FVIIIa/FIXa complex) forming a complex that further activates thrombin. This process is referred to as the thrombin burst [54,55,56,57,58]. Thrombin can be directly inhibited by anti-thrombin III, which also inhibits FXa, FIXa, and FXIIa [59], and thrombin production could be halted by the tissue factor pathway inhibitor (TFPI), which inhibits the TF/FVIIa complex [60]. Finally, and in the presence of calcium, fibrin forms fibrin polymers [61], and thrombin activates FXIII to form FXIIIa, which crosslinks and strengthens fibrin polymers forming a fibrin mesh that strengthens the blood clot [62].

### 3.2. Fibrinolysis, Anti-Coagulation Factors, and Coagulation Inhibitors

Fibrinolytic mechanisms are activated following fibrin mesh formation. Plasminogen, the precursor of plasmin, an anti-coagulation proteolytic enzyme, is activated into plasmin by the urokinase-type plasminogen activator (uPA) and tissue-type plasminogen activator (tPA). PAs have a short half-life in circulation due to the presence of circulating inhibitor plasminogen activator inhibitors (PAIs). Plasmin cleaves fibrin into soluble fibrin degradation products (FDP). Plasminogen and PAs bind FDP exposed carboxy-terminal lysine amino acid, which leads to the activation of plasmin and the cleavage of fibrin to form FDP. Moreover, newly activated plasmin activates PAs by converting single-chain PAs to their two chain counterparts, exerting positive feedback on plasmin activation [63,64,65]. Thrombin activated fibrinolysis inhibitor (TAFI) is another zymogen that is activated by the thrombomodulin (TM)/thrombin complex to form TAFIa [66]. TAFIa downregulates fibrinolysis by removing the C-terminal lysine from the FDP, thus preventing tPA/plasminogen/fibrin complex formation and inhibiting further plasmin activation [66]. It is however worth noting that TM can act as a cofactor for APC activation and upregulate fibrinolysis by attenuation of thrombin production [67,68]. Thus, TAFIa and fibrinolysis regulation are linked to TM levels of the vasculature [69]. Moreover, the inhibition of coagulation is achieved through the presence of circulating protease inhibitors such as anti-thrombin, heparin cofactor II, TFPI, and CI inhibitors. Anti-thrombin targets thrombin, FXa, FIXa, FXIa, and FXIIa, while TFPI targets FXa and the TF/FVIIa complex. Heparin cofactor II and protein C target thrombin [34].

Protein C (PC), a vitamin K-dependent glycoprotein, exerts a pro-fibrinolytic effect by regulating thrombin formation and TAFI activation. Particularly, increasing thrombin formation leads to the binding and activation of PC-forming active protein C APC, secondary to increased thrombin interaction with TM [68]. PC activation is enhanced when bound to the endothelial cell protein C receptor (EPCR) [70]. Following APC formation, APC breaks from the EPCR and TM/thrombin complex, binds to its cofactor protein S (PS), and targets FVa and FVIIIa, thus preventing FXa formation and limiting the thrombin activity [68,71].

### 3.3. Pathophysiology of Coagulopathies

Two common hereditary hypercoagulable conditions include hemophilia and von Willebrand disease. Hemophilia A is the most frequent type of hemophilia and is correlated with FVIII deficiency, while Hemophilia B is correlated with FIX deficiency. Hemophilia C is the least frequent of the three hemophilia subtypes and is correlated with factor XI deficiency [72,73]. Von Willebrand disease is associated with the abnormal production of vWF [74]. Vitamin K is a crucial cofactor for the synthesis and activation of the coagulation factors FII, FIX, and FX, and the anticoagulation proteins (C and S). The lack of Vitamin K leads to the production of under-carboxylated factors that cannot bind to calcium [75].

Hypercoagulable conditions are associated with thrombophilia and are categorized into acquired and inherited conditions [4,76]. The majority of thrombosis is due to thrombophilia, especially in the case of deep vein thrombosis (DVT) [77]. DVT and pulmonary embolism (PE), both known as venous thromboembolism (VTE), are the most frequent conditions correlated with thrombophilia. Major inherited conditions causing thrombophilia include FV Leiden mutation, anti-thrombin III deficiency, PC-PS deficiency, and prothrombin-related thrombophilia [78]. FV Leiden thrombophilia is the most common type of thrombophilia and is associated with the emergence of an APC-resistant FV mutant [79]. Prothrombin-related thrombophilia is the second most common type of thrombophilia and arises from a prothrombin G20210A gene mutation, which enhances DVT occurrence secondary to increased thrombin levels [80]. Anti-thrombin deficiency is also associated with an increased incidence of thrombotic episodes [78,81]. Finally, PC and PS deficiencies enhance the development of DVT and PE [82,83]. There exist two major types of PC deficiencies, type I which is correlated with reduced circulating PC concentrations, and type II which is correlated with a reduction in PC functional activity [84].

## 4. Novel Avenues in Thrombosis Research

In addition to inherent disorders of coagulation, several pathological conditions such as cancer and sepsis are associated with a hypercoagulable state, to which extracellular vesicles significantly contribute. Extracellular vesicles can transfer prothrombotic molecules inter-cellularly and mediate the dissemination of coagulation resulting in arterial and venous thrombosis. As the role of cell-derived and platelet-derived EVs in physiological and pathophysiological states is being continuously revealed, an increased interest in the therapeutic manipulation of these entities is emerging [85]. The cellular release of vesicles is a common and evolutionary-conserved process [86]. The underlying mechanisms of vesicle formation, component sorting, and release have witnessed extensive research within the past decade. Here, we review key concepts of EV biology and thoroughly discuss the implication of cell-derived and platelet-derived EVs in hypercoagulable disease states, especially in cancer and sepsis.

### 4.1. Extracellular Vesicles as Novel Modulators of Coagulation

The term extracellular vesicle denotes numerous subpopulations of cell-shed, non-replicative lipid bilayer-enclosed vesicles [5,6,7]. EV subtypes are characterized by distinct cellular origins and different mechanisms of biogenesis, insights which were provided by means of transmission and immune-electron microscopy. EVs are widely distributed in human bodily fluids including urine, blood, saliva, and synovial and cerebrospinal fluids [86]. Advances in the standardization of EVs isolation and characterization are comprehensively reviewed elsewhere [5,86]. EVs, which include MVs, exosomes, membrane particles, and apoptotic bodies [8], have attracted significant interest as they are implicated in the regulation of coagulation physiology and the pathophysiology of hypercoagulable states. Although early observations highlighted procoagulant properties of EVs in healthy individuals by supporting low-grade thrombin formation [86], emerging evidence supports a fibrinolytic rather than a procoagulant activity of EVs in healthy humans [87].

Procoagulant EVs are released by a plethora of cells including endothelial cells, adipocytes, and macrophages among others implicating these entities in the pathogenesis of cardiovascular diseases and their associated procoagulant states as summarized in Table 1. [88,89,90,91]. The concentration of EVs in healthy individuals amounts to 10^3^–10^11^ per mL depending on the methodologies used for EV quantification and their respective sensitivities [92,93]. The abundance of circulating EVs was shown to regress in an age-dependent manner [94]. It was, however, demonstrated that endothelial EVs from elderly subjects maintain a procoagulant activity contrary to that of young control subjects [95]. Moreover, increased EV plasma levels are observed in diseases associated with a procoagulant state such as thrombotic thrombocytopenic purpura [96], sickle cell disease [97], and heparin-induced thrombocytopenia [98]. Enhanced circulating EV levels are also observed in established cardiovascular diseases including acute coronary syndrome [99] and stroke [100,101], but also in metabolic disorders predisposing to cardiovascular dysfunction such as type 2 diabetes and metabolic syndrome [102,103,104]. Recent evidence suggests that cellular-derived and platelet-derived EVs modulate thrombogenicity in patients with atrial fibrillation [105]. AF is associated with elevated circulating levels of platelet-derived and mononuclear cell-derived EVs and reduced endothelial cell-derived EVs [105]. Elevated EVs including procoagulant TF-expressing, P-selectin-expressing, or PS-expressing EVs were demonstrated in AF patients and were suggested to contribute to AF-associated thrombogenicity [106,107,108]. Whether this increase in EVs concentration associated with these disorders drives their pathogenesis or is secondary to disease development requires further investigation. Importantly, the mechanisms by which coagulation factors modulate the release of EVs remain largely unknown. Nevertheless, recent evidence implicates FVIIa/EPCR/PAR1-mediated signaling in the induction of the release of procoagulant EVs from endothelial cells primarily through a ROCK-dependent pathway [88,89].

**Figure 2:**
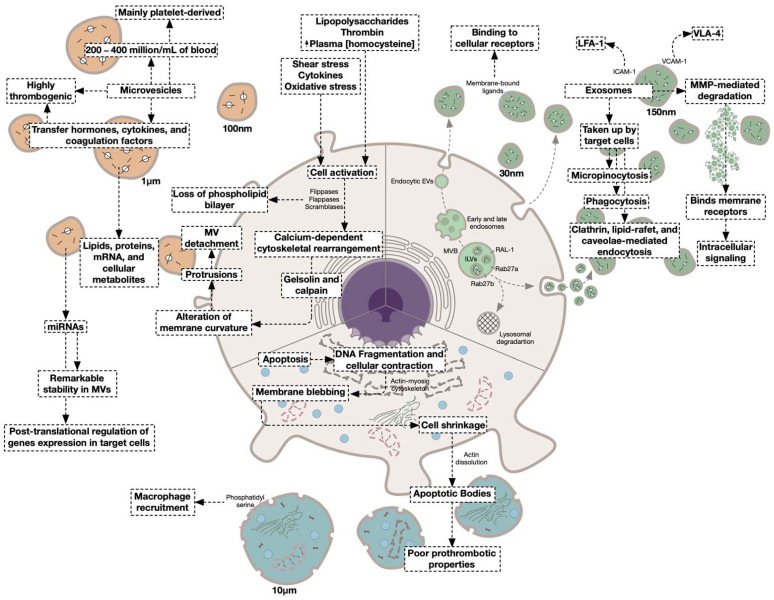
General mechanisms governing the formation and secretion of extracellular vesicles including microvesicles, apoptotic bodies, and exosomes. Microvesicles are 100 nm–1 μm in size and are secreted following cellular activation. Several stimuli promote microvesicle formation including shear stress, oxidative stress, several cytokines, as well as lipopolysaccharides, thrombin, and high plasma levels of homocysteine. Following cellular activation, flippases, floppases, and scramblases mediate the loss of the phospholipid bilayer integrity. Simultaneously, calcium-dependent, gelsolin and calponin-executed cytoskeletal rearrangement occur resulting in the alteration of the membrane curvature. This allows for the emergence of plasma membrane protrusions and the detachment of MVs. MVs contain several molecules such as miRNAs, lipids, proteins, and cellular metabolites that potentially modulate key functions in target cells. Apoptotic bodies, which have poor procoagulant activity, are released from apoptotic cells following membrane blebbing. Exosomes originate from an endocytotic extracellular vesicle that passes through the endosomal pathway, leading to the formation of multi-vesicular bodies. Exosomes are released from multi-vesicular bodies where they interact with target cells either by binding their surface receptors or through exosome-carried molecules following exosomal degradation. EV, extracellular vesicle; ICAM-1, intracellular adhesion molecule-1; LFA-1, leukocyte function-associated antigen-1; miRNA, micro RNA; MMP, matrix metalloproteinase; MV, microvesicle; RAL-1, Ras-related GTPase homolog; VCAM-1, vascular cell adhesion molecule-1; VLA-4, very late antigen-4.

EVs represent a novel mechanism in intercellular communication in both physiological and pathophysiological states [109], carrying a cargo of bioactive molecules, such as lipids, proteins, messenger RNAs (mRNA), cellular metabolites, DNA, and non-coding RNA molecules including microRNAs (miRs), long non-coding RNAs (lncRNAs), and circular RNAs (circRNAs) into target cells (Figure 2) [110,111]. Particularly, intact EV-enclosed non-coding RNA molecules can be isolated from the circulation despite the presence of significant RNase activity [112,113]. Indeed, EVs enclosing miRNAs have been demonstrated to participate in the modulation of various diseases as miRNAs regulate key factors of hemostasis [114]. It is worth mentioning that platelets inherit a diverse array of coding and non-coding sequences and translational machinery from megakaryocytes and represent a major source of circulating miRs [115]. Nevertheless, the contribution of activated platelet-derived EVs to the pool of circulating non-coding RNAs requires further investigation and surely adds to the complexity of studying thrombosis-associated EV-encapsulated non-coding RNAs.

**Table 1:** Cardiovascular diseases are associated with increased levels of procoagulant circulating extracellular vesicles. Clinical evidence implicates procoagulant cellular-derived and platelet-derived extracellular vesicles in the promotion of cardiovascular disease-associated thrombogenicity. EVs, extracellular vesicles; MVs, microvesicles; PS, phosphatidylserine; TF, tissue factor.

Disease	Alteration of the Abundance of Circulating Procoagulant Extracellular Vesicles	References
Thrombotic thrombocytopenic purpura (TTP)	Increased levels of circulating platelet-derived EVs	[96]
Idiopathic thrombocytopenic purpura (ITP)	Increased levels of circulating platelet-derived EVs	[116]
Heparin-induced thrombocytopenia (HIT)	Increased levels of circulating TF- expressing platelet-derived EVs	[98,117]
Sickle cell anemia	Elevated circulating levels of erythrocyte, platelet, monocyte, and endothelial cell-derived EVs	[93,118,119]
Disseminated intravascular coagulation (DIC)	Increased levels of circulating endothelial cell-derived EVs (suggested as a biomarker of DIC caused by septic shock)	[120,121,122]
Acute coronary syndromes (ACS)	Elevated platelet and monocyte-derived MVs Increased levels of circulating CD31^+^ CD42b^−^ MVs	[99,123,124]
Venous thromboembolism (VTE)	Elevated levels of circulating endothelial cell and platelet-derived PSGL-1 and CD62P-expressing MVs	[125,126]
Acute ischemic stroke (AIS)	Elevated levels of circulating endothelial cell-derived MVs	[127]
Paroxysmal nocturnal hemoglobinuria (PNH)	Increased levels of circulating platelet, monocyte, and endothelial cell-derived EVs	[128,129]
Coronary heart disease (CHD)	Elevated levels of CD31^+^, CD42^−^, and CD144^+^ endothelial cell-derived EVs	[124]
Acute myocardial ischemia	Elevated levels of circulating CD66b^+^, CD62E^+^, and CD142^+^ EVs	[130]
ST-segment elevation myocardial infarction (STEMI)	Elevated levels of circulating leukocyte-derived CD11^+^, endothelial cell-derived CD105^+^, and TF-bearing MVs Increased levels of erythrocyte-derived but not platelet-derived MVs	[131]
Acute stroke (AS)	Elevated levels of circulating CD62E^+^ endothelial cell-derived EVs	[101]
Acute pulmonary embolism (APE).	Increased levels of circulating TF-expressing MVs	[132,133]
Atrial Fibrillation (AF)	Increased levels of circulating platelet-derived and mononuclear cell-derived EVs and reduced levels of circulating endothelial cell-derived EVs Increased levels of circulating procoagulant EVs expressing TF, PS, and P-selectin	[105,106,107,108]

#### 4.1.1. Microvesicles

First documented in the late 1960s and thought to be “cell dust”, microvesicles, also known as ectosomes or microparticles, are lipid bilayer-enclosed sacs ranging in diameter between 50 nm to 1.0 μm [8,134]. Although initially thought to be plasma membrane fragments released from platelets as part of the coagulation process [135,136], MVs are now believed to be important players in intercellular communication. Importantly, MVs were shown to transfer bioactive molecules such as hormones, cytokines, and coagulation factors, and are implicated in the modulation of coagulation processes [135]. Indeed, it was demonstrated that neutrophils and monocytes release TF-expressing MVs in circulating blood near the surface of platelets [137]. Two different mechanisms elicit MVs formation, apoptosis, and cell activation. During apoptosis, DNA fragments and the cell contracts resulting in an actin-myosin cytoskeleton-mediated membrane blebbing [138]. These blebs are distinct from those generated through cellular activation in size and protein composition. As the bleb formation continues, cells shrink into small apoptotic bodies as polymerized actin undergoes dissolution (Figure 2). The shedding of MVs can also occur in an apoptosis-independent manner following the exposure to certain physiological and pathophysiological stimuli among which are stimulation by proinflammatory cytokines, ROS, PMA, LPS, complement C5b-9, PAI-1, thrombin, and collagen [139,140]. It is worth mentioning that the mechanism by which cellular-derived and platelet-derived MVs release is induced determines their phenotype and function. Upon cellular activation, MVs are released in a calcium-dependent mechanism which triggers dynamic cytoskeletal rearrangement of the plasma membrane mediated by a consortium of enzymes including flippases, floppases, and scramblases [8]. Calcium also activates enzymes that modulate the cytoskeleton and promote the disassembly of sub-plasma membrane-localized actin such as gelsolin and calpain. All these changes in the structure and dynamics of the plasma membrane result in alterations of the membrane curvature and the formation of protrusions allowing the MVs to detach [141]. Dramatic extremes of temperature reduce the cellular capacity to internalize EVs and were shown to abolish the procoagulant activity of endothelial cell-derived MVs [142,143]. Moreover, calcium chelation, formalin fixation, and cytoskeleton disruption have been shown to reduce EVs cellular uptake [143].

The concentration of MVs in plasma has been estimated at 2–4 × 10^8^ per mL [135]. Although the majority of these MVs are platelet-derived [144,145], MVs deriving from granulocytes, ECs, monocytes, and erythrocytes have been identified in states of health and disease. Indeed, human blood enrichment with circulating MVs from healthy subjects enhances platelet adhesion and fibrin deposition on the damaged porcine aorta and human atherosclerotic arteries [146]. Additionally, circulating thrombin-generating cell-derived MVs occur in healthy subjects and their procoagulant effects occur through TF-independent pathways and were inhibited, at least in part, through FXII, FXI, or FVIII-targeting antibodies [86]. Platelet-derived MVs express TF and contain procoagulant membrane components that potentiate coagulation due to their unusual lipid arrangements, including the externalization of phosphatidylserine (PS) [147,148,149]. Defects in systemic PS-expressing MVs clearance were found to instigate hypercoagulation in lactadherin-deficient mice [142,150,151,152]. TF-exposing MVs were shown to derive from platelets, neutrophils, erythrocytes, granulocytes, and monocytes, while their capacity to induce thrombin generation was reduced following the incubation of TF-neutralizing antibody [153]. In addition to TF and TFPI, other procoagulant molecules and receptors carried by MVs include P-selectin glycoprotein ligand-1 (PSGL-1), which mediates the interaction between MVs and CD62P expressed on activated platelets and endothelial cells [154]. Moreover, an increased number of circulating MVs was demonstrated in patients with vascular inflammation and heart failure, stroke, and myocardial infarction, at least in part secondary to platelet activation [124,150,151,155,156,157]. The abundance of circulating procoagulant MVs is also increased in ACS, chronic ischemic heart disease patients, CAD, heart failure, as well as atrial fibrillation patients, and following vascular stent implantation [130,151,157,158,159,160]. Particularly, increased procoagulant MVs in CAD and ACS are suggested to be endothelial cell-derived [99,124,161]. Indeed, the procoagulant potential of monocyte-derived MVs is the highest, followed by that of endothelial cells, platelets, and erythrocytes-derived MVs [162]. Indeed, several studies have shown that TF-expressing MVs derive from monocytes [163,164,165], while platelet-derived MVs were shown not to support FXa generation and to exhibit reduced procoagulant activity [166]. Furthermore, emerging evidence suggests that cellular phenotypes and polarization, such as the proinflammatory and anti-inflammatory polarization modalities of macrophages, modulate the procoagulant potential of their secreted MVs [167].

#### 4.1.2. Platelet-Derived Microvesicles

**Figure 3:**
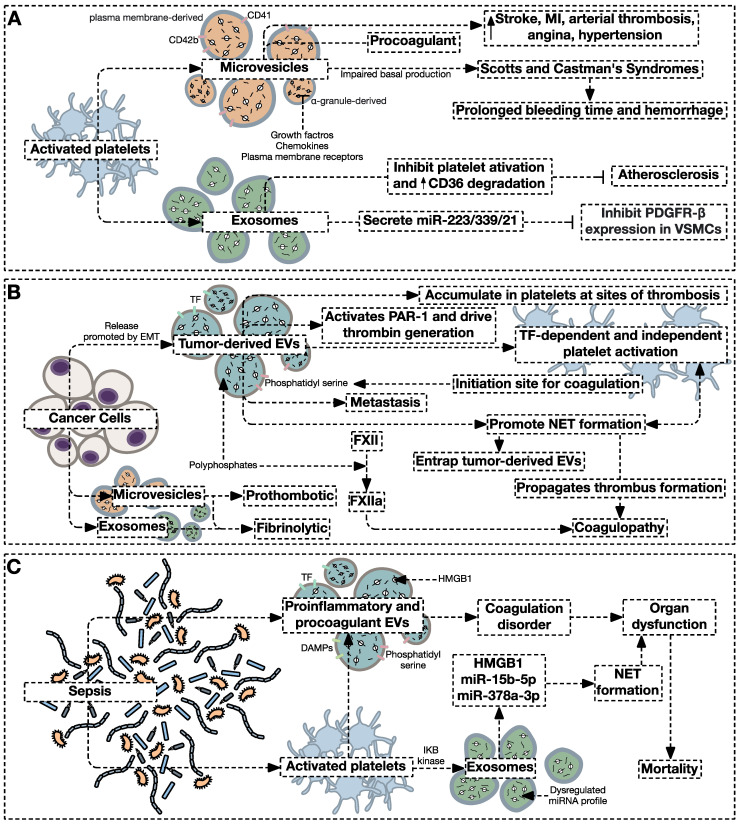
General pathways through which extracellular vesicles participate in disease-associated procoagulant states. (**A**) Activated platelets secrete procoagulant microvesicles that increase in different cardiovascular disorders. Impaired production of platelet-derived microvesicles was shown to participate in different bleeding disorders such as Scott’s and Castman’s Syndromes. On the other hand, activated platelet-derived exosomes are thought to counteract the progression of atherosclerosis. (**B**) Cancer cell-derived extracellular vesicles are procoagulant through different pathways that initiate TF-dependent or independent platelet activation leading to disseminated coagulopathy. (**C**) Sepsis causes end-organ damage mainly through activating the coagulation system. In sepsis, proinflammatory and procoagulant EVS are released into the circulation, while procoagulant exosomes are released for activated platelets, where both participate in sepsis-associated mortality.

Platelet-derived MVs can be distinguished by their expression of the surface markers CD41 and CD42b [168,169]. Platelet-derived MVs encompass a broad vesicle size range depending on their content of growth factors, chemokines, and plasma membrane receptors [170]. It is suggested that smaller MVs originate from α-granules, while larger ones derive from the plasma membrane. It was demonstrated that the number of MVs released from activated platelets does not match the number of MVs released by platelets in healthy subjects in vivo. This led scientists to theorize that MVs are derived from circulating quiescent platelets following shear stress exposure [171]. However, it is now hypothesized that CD41^+^, filamin-A-expressing MVs continuously derive from megakaryocytes and simultaneously exist with regular platelet-derived MVs in healthy humans [172]. Importantly, CD41^+^ MVs were shown not to be associated with an increased risk of CVDs, which reinforces the speculation that the non-active form of MVs possibly derives from megakaryocytes [173,174,175]. Nevertheless, it is suggested that following the activation of platelets, increased levels of platelet-derived MVs are released as depicted in Figure 3 [176]. Indeed, platelets activated by ADP, thrombin, and collagen were shown to release an enhanced number of MVs [177,178,179,180,181]. Anti-coagulant drugs including clopidogrel, which targets adenosine P2Y12 ADP receptor or ticagrelor, or the antiplatelet aspirin reduce the release of procoagulant platelet-derived EVs [182,183,184]. Direct thrombin inhibitors including dabigatran and melagatran reduce TF-expressing platelet-derived MVs release in response to thrombin or ADP stimulation [185]. Additionally, monoclonal antibodies targeting GPIbαinhibit procoagulant platelet-derived MVs release under high shear stress more efficiently than does the GPIIb/IIIa antagonist abciximab [186]. Moreover, activated platelet-derived MVs are 50–100 fold more procoagulant than the negatively-charged surface of platelets [187].

Although a balanced production of platelet-derived MVs is pivotal to the induction of angiogenesis and endothelial cell proliferation in vivo [175,188], aberrantly low or high production of platelet-derived MVs has been demonstrated in several pathological states. Individuals with Scott’s Syndrome, Glanzmann’s thrombasthenia, and Castaman defect have an impaired basal capacity to generate MVs resulting in an enhanced susceptibility for hemorrhage [189,190,191,192]. Alternatively, elevated levels of platelet-derived MVs were demonstrated in several thrombosis-associated diseases including arterial thrombosis [189], sickle cell disease [97], and CVDs such as angina and hypertension [189]. It has also been suggested that platelet-derived MVs transfer miRs to target cells as a form of intercellular communication [193,194]. For example, the transport of miRNA-133a, whereby levels are significantly elevated in patients with acute MI and angina pectoris occurs via MVs reaching target cells in the heart as well as other organs following these cardiovascular events [195,196]. Moreover, miR-containing platelet-derived MVs function beyond coagulation. For example, platelet-derived miR-1915-3p-containing MVs suppress the expression of Rho GTPase family member B, inducing megakaryopoiesis [197].

##### Exosomes

Exosomes are nano-sized particles that range in diameter between 50 and 150 nm and are secreted by almost all mammalian cells [85,198,199]. Exosomes’ protein content is characterized by a consortium of transmembrane and non-membrane bound proteins including Annexin V, GTPases, Rab, and flotillin, as well as protein components of the endosomal sorting complexes required for transport (ESCRT), such as AGLT-2-interacting protein X (Alix), heat shock proteins (HSPs), tumor susceptibility gene 101 (TSG101), tetraspanins (CD63, CD81, and CD9), and integrins (e.g., α4β1 and VCAM-1 on endothelial cells and αLβ2 on leukocytes) [8,200,201,202,203]. As such, patient treatment with abciximab, which targets the integrin αIIβ3, inhibits the uptake of PS-expressing EVs by human endothelial cells [204].

Exosomes derive from the fusion of the plasma membrane with the endosomal network. However, the mechanisms of exosome biogenesis, formation, and release are quite complicated, and only partly unraveled [8,205]. It has been proposed that exosomal biogenesis starts with the formation in specialized regions of the plasma membrane of endocytic EVs, which is followed by the formation of intraluminal vesicles (also called exosome precursors) within multi-vesicular bodies. Intraluminal vesicles then detach from the plasma membrane and move to the early endosome, where they fuse. In this compartment, internalized membrane receptors undergo conformational changes and dissociate from their ligands. The dissociated ligands are destined for degradation after their sorting into lysosomes. Alternatively, internalized receptors and retained ligands can be recycled to the plasma membrane or transferred to late endosomes. In late endosomes, proteins are either degraded through the lysosomal pathway or are incorporated in intraluminal vesicles, which upon budding from multi-vesicular bodies, are conveyed to the plasma membrane and are released (Figure 2) [8,206]. It is worth noting that the inhibition of exosome biosynthesis and release in senescent cells promoted DNA damage and accelerated cellular apoptosis-like cell death [207], suggesting that exosome-mediated release of detrimental DNA fragments as well as other cellular components during cellular senescence may play a protective role preventing the development of innate immune responses.

Accumulating evidence suggests the implication of various molecules in the membrane fusion and release of exosomes. Small Rab GTPases were shown to drive exosome secretion, while Rab27a and Rab27b are implicated in multi-vesicular body docking to the plasma membrane [208]. Multi-vesicular body formation and exosome secretion are also controlled by Ras-related GTPase homolog (RAL-1) [209]. Following secretion, circulating exosomes can be taken up by target cells by three different mechanisms; fusion, binding, and endocytosis [143]. In that sense, exosomes expressing membrane-bound ligands may bind to receptors of target cells to activate intracellular signaling [210]. For example, the expression of intracellular adhesion molecule-1 (ICAM-1) and vascular cell adhesion molecule-1 (VCAM-1) on the surface of EVs allows their binding to recipient cells through receptor interactions with leukocyte function-associated antigen-1 (LFA-1) and very late antigen-4 (VLA-4), respectively [211,212]. Moreover, proteoglycans such as heparan sulfate proteoglycan receptors and glycoproteins such as the hyaluronan receptor CD44, or lectins present other protein groups implicated in the uptake of EVs by recipient cells [139]. Various mechanisms of endocytosis, being the major type of exosome uptake, exist and include micropinocytosis, phagocytosis, clathrin-mediated and clathrin-independent endocytosis, lipid raft-mediated endocytosis, and caveolae-mediated endocytosis [143,213]. As such, inhibiting clathrin-dependent endocytosis in endothelial cells reduces the uptake of PS-expressing EVs [204]. Moreover, it was demonstrated that inhibiting caveolae endocytosis, either pharmacologically or through knocking down caveolin-1, as well as cholesterol depletion, reduces exosomes and MVs uptake by target cells [163]. It is proposed that exosomes actively contribute toward a procoagulant phenotype through carrying key effectors of coagulation such as fibrinogen [214]. Nevertheless, exosomes were demonstrated to be less efficient in promoting coagulation than MVs [215].

#### 4.1.3. Platelet-Derived Exosomes

Platelet-derived exosomes were shown to suppress athero-thrombotic processes by enhancing the proteolytic degradation of CD36 and by inhibiting platelet activation and subsequent thrombosis [216]. It was also shown that thrombin-activated platelets secrete miR-223 during inflammation, which downregulates the expression of ICAM-1 in endothelial cells [217]. Moreover, exosomes derived from thrombin-stimulated platelets contain miR-223, miR-339, and miR-21 and inhibit the expression of platelet-derived growth factor receptor-β in VSMCs [218]. In an miR-223-deficient mouse model of carotid thrombosis, prolonged times to occlusive thrombosis were observed demonstrating a protective effect of miR-223 deficiency [219]. The transfusion EVs from WT mice to miR-223-deficient mice reversed the reduction in thrombosis time. Another study suggested that platelet miR-223 levels were significantly lower in hypertensive patients and these levels were suggested as prognostic markers for cardiovascular disease in these patients [220]. Moreover, miR-233 platelet levels were elevated while their content in platelet-derived MVs decreased in a hypertensive, hyperlipidemic animal model of atherosclerosis [221]. This indicates an active mechanism of selective miR packing into platelet-derived MVs in different pathologies, which provides novel targets for the treatment of cardiovascular diseases. It was also demonstrated ex vivo that platelets support gymnosis, that is, the internalization of ectopic miRs in absence of conventional transfection reagents and their incorporation into RNA-induced silencing complexes (RISCs) by endocytic pathways [222]. The uptake of miR-223-3p by thrombin or fibrinogen-induced platelets inhibited exosome and MVs generation [222]. A recent study demonstrated that senescent platelets in vitro include miRNAs relevant to atherosclerosis and inflammation [223]. Moreover, senescent platelet-derived EVs contain upregulated levels of miRs involved in obesity, diabetes, and metabolic and vascular diseases (Figure 3) [223]. Although the multifaceted roles of EVs in atherosclerosis development and progression are appreciated [224,225], further investigation into the possible roles of platelet-derived exosomes in driving the pathogenesis of coagulopathies is warranted.

#### 4.1.4. Apoptotic Bodies

Apoptotic bodies are large, permeable vesicles released following cellular apoptosis, with sizes ranging from 50 nm to 5.0 μm in diameter [150,226]. Importantly, apoptotic bodies are less regular in shape in comparison to MVs and are released in advanced stages of apoptosis as a result of cellular contractions and the increased hydrostatic pressure in dying cells [227,228]. Some MVs may also be released during this stage of membrane blebbing. These bodies can contain various cellular contents, cellular organelles, and DNA fragments and can thus participate in processes ranging from tissue regeneration to horizontal DNA transfer. For example, the DNA content of endothelial cell-derived apoptotic bodies was shown to promote the proliferation and differentiation of human endothelial progenitor cells [229]. Similarly, macrophage-derived apoptotic bodies were shown to contain miR-221/222 which promotes the proliferation of lung epithelial cells [230]. Fibroblast-derived apoptotic bodies were also demonstrated to contain amino acids that induce gene recombination in target cells [231]. Apoptotic bodies are cleared through phagocytosis by cells of the innate immune system and endothelial cells [232,233]. In comparison with MVs, apoptotic bodies have poor prothrombotic properties and were suggested to use phosphatidylserine for the recruitment of phagocytes to sites of cellular death rather than for platelet activation (Figure 2) [234]. Nevertheless, PS-expressing apoptotic EVs were shown to directly associate with FXII and support thrombin generation [149]. As such, the procoagulant roles of apoptotic bodies are yet to be elucidated and require further investigation.

### 4.2. Extracellular Vesicles as Drivers of Hypercoagulable States

#### 4.2.1. Extracellular Vesicles in the Cancer-Associated Prothrombotic States

A significantly higher risk for the development of venous thrombosis in cancer patients is well documented [235]. Cancer-associated thrombosis is a multi-factorial process in which several mechanisms are implicated, the molecular underpinnings of which are still obscure [236]. Several studies highlighted a role for tumor-derived EVs expressing TF and PS in the thrombotic manifestations of many cancers including multiple myeloma, and breast and prostate cancers [237,238,239]. Indeed, procoagulant, PS-expressing tumor-derived EVs provide initiation sites for blood coagulation and tumor-derived EVs may activate platelets and promote their aggregation through both TF-dependent and TF-independent mechanisms [109,240,241,242]. TF-expressing EVs were first isolated from plasma samples of advanced colorectal cancer patients [243] and were then detected in the plasma of pancreatic, lung, ovarian, colorectal, prostate, and breast cancer patients [244,245,246,247]. Moreover, one study showed that tumor-derived TF-expressing EVs induce DVT and that these EVs cooperate with host TF in order to cause the prothrombotic state in pancreatic cancer [248].

Emerging evidence provides conflicting perspectives on the implication of tumor-derived and platelet-derived procoagulant EVs in the pathogenesis of cancer-associated thrombosis and their use as biomarkers predicting coagulopathy [249,250,251,252,253,254,255]. Whereas a strong correlation between TF-expressing EVs and VTE was demonstrated in patients with pancreatic cancer [256,257], other studies in different cancers failed to arrive at similar associations [256,258,259,260,261]. Particularly, a prospective cohort study demonstrated an association between MVs TF activity in cancer patients and total mortality and prognosis, but not thrombosis [260]. These conflicting outcomes probably arise from the differential use of various techniques for TF measurement, different EV purification methods, and variable antibody and assay sensitivity. As novel assays measuring the activity of TF in plasma EVs are developing, a better comprehension of the association between TF-expressing EVs and cancer-associated thrombosis will become possible. It is noteworthy that although cancer cell-derived exosomes and MVs display fibrinolytic activities, only cancer cell-derived MVs display prothrombotic activities [262]. Importantly, neither exosomes nor MVs displays fibrinolytic activity under physiological conditions.

As vascular and tumor cell-derived, TF-bearing EVs were shown to drive coagulopathy [263,264], it was shown that TF-expressing EVs promote prothrombotic states in murine models of Lewis lung carcinoma, melanoma, pancreatic cancer [200,264,265,266,267]. Mice bearing TF-positive tumors and elevated levels of circulating TF-expressing EVs were shown to exhibit enhanced thrombosis [268]. Mice injected with TF-overexpressing ovarian cancer cells exhibited enhanced venous thrombus formation following experimental stenosis [269], while mice bearing tumors under-expressing TF exhibited halted coagulation, platelet aggregation, and thrombus formation [270]. Coculturing prostate cancer cells with high TF expression with peripheral blood mononuclear cells and platelets enhanced EVs TF activity while no such observation was shown in prostate cancer cells with low TF expression [247]. It was also suggested that glioma-derived podoplanin and TF coexpressing EVs cooperatively enhance micro-thrombosis in glioma xenograft murine models [271]. It is worth noting that the procoagulant potential of TF-expressing MVs derived from human tumor cell lines is reduced in PAR2^−/−^ mice following clopidogrel treatment. Moreover, recent evidence suggests that cancer cell-derived EVs expressing polyphosphates induce blood coagulation by activating FXII, with pancreatic and lung cancer cell lines exhibiting the most potent activity [272,273]. Degrading EV’s polyphosphates inhibits FXII activation [273] while blocking the polyphosphate/FXII pathway protects against thromboembolism without promoting hemorrhage in mice [274]. It was therefore suggested that the polyphosphate/FXII axis could be targeted to decrease cancer-associated thrombosis [275].

Moreover, cancer cells and their secreted EVs promote the formation of neutrophil extracellular traps (NETs), which in addition to modulating cancer growth and metastasis, contribute to cancer-associated coagulopathy [109,276,277,278,279]. NETs represent a molecular trap that is formed by DNA chromatin, histones, and serine proteases in addition to other components, and these components, rather than intact NETs are suggested to drive NETosis-associated coagulopathies [280,281]. Peptidylarginine deiminase 4 (PAD4), an enzyme that mediates chromatin decondensation, was shown to regulate NETosis and coagulopathy where PAD4^−/−^ mice exhibit impaired NETs formation [282,283,284,285]. While P-selectin was shown to promote NETosis through binding to PSGL-1 [286], fibrin and APC were shown to inhibit NETosis [287,288]. Activated platelets promote NETs formation, which forms a viscous cycle that propagates thrombus formation [289,290,291].

NETs are present in both arterial and venous thrombi of both human and experimental mice and were shown to promote platelet activation and aggregation in histone-dependent and independent pathways [289,292,293,294]. Increased markers of NETs formation were also detected in cancer patients and were associated with an increased risk of thrombosis [295,296,297,298,299]. Interestingly, NETs can entrap tumor-derived pro-coagulant EVs which amplifies the establishment of the prothrombotic state [266,295]. Indeed, mice orthotopically injected with 4T1 breast cancer cells exhibit enhanced exosome-induced NETs formation, which is associated with enhanced thrombosis [295]. Targeting NETs exerted an antithrombotic effect which further stresses the importance of NETs generation on thrombus formation [283,289,295]. Epithelial-to-mesenchymal transition, another hallmark of cancer, may also promote the release of TF-bearing EVs from tumor cells [263]. Although tumor-derived EVs activate platelets, the platelets play a fundamental role in accumulating these vesicles at the site of thrombosis through PSGL-1 in an EVs membrane integrins-dependent manner [266,270].

#### 4.2.2. Extracellular Vesicles in the Sepsis-Associated Prothrombotic States

Sepsis is a major cause of thrombosis and organ failure with a mortality rate approaching 50%, where early diagnosis and treatment are pivotal for patient survival. Elevated levels of circulating procoagulant and proinflammatory EVs were documented in sepsis and were suggested to contribute to coagulation disorders and organ dysfunction and are correlated with mortality [300,301,302,303,304,305,306]. Several factors expressed on the vesicle surface including TF and PS, and bioactive substances contained in vesicles such as nucleosomes and high-mobility group box 1 (HMGB1), contribute to this prothrombotic state [307,308,309]. Although platelet-derived EVs represent the major population of EVs in sepsis [310,311], endothelial cells, monocytes, neutrophils, and red blood cells were extensively studied and shown to participate in TF-positive EV expression. Therefore, further investigation is still required to delineate the exact cellular source of septic coagulopathy [312,313].

Although the procoagulant role of these EVs in sepsis is starting to unveil, the lack of standardized assays and procedures for the characterization of these vesicles in sepsis yielded inconclusive results [312]. The upregulation of TF expression in EVs was evident in EVs released from activated monocytes [314], endothelial cells, and platelets, and predicts the severity of sepsis [315,316,317]. Likewise, Zhang et al. reported that endothelial cells treated with EV-containing serum from septic patients demonstrated increased levels of exposed PS in comparison to controls [318]. In another study, Tripisciano et al. induced the generation of thrombin in vesicle-free human plasma to which platelet-derived EVs were added [319]. Importantly, this was abrogated by the PS-binding annexin V but not by anti-TF antibodies. In sepsis, damage-associated molecular patterns (DAMPs) are released from activated and damaged cells and are transferred by EVs [320]. Platelet-derived EVs were shown to preferentially associate with circulating monocytes, contributing to sepsis-associated prothrombotic state [321,322].

Exosomes play an important role in coagulation and inflammation processes as well as organ dysfunction in sepsis. It was suggested that elevated exosome plasma level predicts the severity of organ failure and mortality in septic patients [323]. Moreover, another study demonstrated a differential, dysregulated miR profile in serum exosomes, total serum, and platelets of sepsis patients [324]. This compartment-specific expression of sepsis-associated miRs, especially in exosomes, was suggested as a novel biomarker in sepsis [325]. Platelets also activate NET formation during sepsis. Treating neutrophils with septic shock patient-derived exosomes significantly increased NET components and correlated positively with disease severity and outcome [326]. This observation was also confirmed in an in vivo model of sepsis. Mechanistically, exosomal HMGB1, miR-15b-5p and miR-378a-3p induced NET formation through the Akt/mTOR pathway [327]. It was also suggested that IκB kinase controls the secretion of platelet-derived exosomes in sepsis [327].

## 5. Conclusions and Future Perspectives

Major advances in our comprehension of coagulation pathways and hemostasis have evolved since the early observations of Marcello Malpighi in the seventeenth century. As hemostasis is defined through the balance of procoagulant and anticoagulant mechanisms, a tilted balance towards either mechanism results in pronounced diseases such as hemorrhage and thrombosis, the magnitude of which depends on the scale of balance disruption. Extracellular vesicles represent an exciting novel avenue in thrombosis research as cellular and platelet-derived extracellular vesicles mediate, at least in part, the procoagulant phenotype observed in cardiovascular disorders, cancer, and sepsis. Indeed, clinical and experimental evidence demonstrates that the procoagulant activity of these EVs extends well beyond their exposure to TF or PS, but encompasses cellular components implicated in the multiplication of coagulation surfaces and intercellular cross-linking at sites of vascular injury. Their pervasive presence in all body fluids including blood and urine allows for their promising utilization as fluid biopsies for the diagnosis and prognosis of diseases in which EVs abundance and content are altered.

Nevertheless, it should be noted that clinical evidence more often than not derives from studies employing relatively small patient cohorts, thus, validation in larger cohorts of patients is warranted. Additionally, distinct methodologies utilized in the isolation and characterization of EVs subtypes such as differential ultracentrifugation, size exclusion chromatography, and density gradient chromatography, yield inconsistent EV preparations with differential purity and composition, halting the collective translational potential of these clinical findings. It is therefore of utmost importance that future endeavors into characterizing the hemostatic roles of EVs occur in accordance with published recommendations and guidelines [5]. Another limitation is presented by our incomprehensive knowledge regarding the effects of concurrent antiplatelet and anticoagulant treatments on the abundance of cellular-derived and platelet-derived EVs and the alteration in their contents. Indeed, further investigation into these research avenues is warranted.

Promising evidence implicates EVs as a drug delivery system and the modulation of their cellular uptake as an avenue in the design of future therapeutics [327]. As such, EVs can be genetically engineered to display antithrombotic, fibrinolytic, or regenerative properties achieving targeted in vivo effects with minimal off-target uptake [328,329]. Nevertheless, and before these applications become available for clinical trials, assessments of their limitations such as cytotoxicity, uptake kinetics, and half-life are warranted [330,331]. Given the overwhelming evidence implicating EVs in the modulation of hemostatic processes, as biomarkers in procoagulant disorders, and as potential drug delivery systems, it becomes evident that advancing our knowledge concerning the mechanisms underpinning EV formation and their pathological relevance will enhance their translation into clinical practice.
